# The neighborhood food environment modifies the effect of the 2009 WIC food package change on childhood obesity in Los Angeles County, California

**DOI:** 10.1186/s12889-020-08779-2

**Published:** 2020-05-13

**Authors:** Christopher E. Anderson, Catherine M. Crespi, May C. Wang, Shannon E. Whaley, M. Pia Chaparro

**Affiliations:** 1 Department of Epidemiology, School of Public Health and Tropical Medicine, Tulane University, 1440 Canal St., Suite 2000, New Orleans, LA 70112 USA; 2 Department of Biostatistics, Fielding School of Public Health, University of California Los Angeles (UCLA), 650 Charles E. Young Dr. South, Box 951772, Los Angeles, CA 90095 USA; 3 Department of Community Health Sciences, Fielding School of Public Health, University of California Los Angeles (UCLA), 650 Charles E. Young Dr. South, 26-051B CHS, Los Angeles, CA 90095 USA; 4 Public Health Foundation Enterprises (PHFE) WIC, 12781 Schabarum Ave, Irwindale, CA 91706 USA; 5 Department of Global Community Health and Behavioral Sciences, School of Public Health and Tropical Medicine, Tulane University, 1440 Canal St., Suite 2200, New Orleans, LA 70112 USA

**Keywords:** Food environment, Nutrition assistance program, Obesity, Nutrition policy, Effect modification

## Abstract

**Background:**

Food packages provided by the Special Supplemental Nutrition Program for Women, Infants and Children (WIC) were revised in 2009 to better align them with the Dietary Guidelines for Americans. This study was conducted to evaluate whether the effect of the food package change on childhood obesity varied by the food environment in the neighborhoods where WIC-participating children live.

**Methods:**

Administrative data from participating children in Los Angeles County, California (2003–2016) were merged with geocoded food vendor information by neighborhood of residence. Obesity risk at age 4 was compared between children receiving old (2003–2009) and new (2010–2016) WIC food packages using sex-stratified Poisson regression models, with interaction terms between WIC package and neighborhood density (number per square mile) of healthy and unhealthy food outlets.

**Results:**

The new food package was associated with a significant decrease in obesity risk. Among boys, the new food package was associated with 8 to 18% lower obesity risk at all healthy and unhealthy food outlet densities, and the association was not modified by neighborhood food outlet density. Among girls, the association of the new food package with obesity risk was protective in neighborhoods with high healthy and low unhealthy food outlet densities, and adverse in neighborhoods with high unhealthy and low healthy food outlet densities. The effect of the new food package among girls was modified by unhealthy food outlet density, with significantly smaller (*p*-value = 0.004) decreases in obesity risk observed in neighborhoods with higher unhealthy food outlet density.

**Conclusions:**

The impact of the food package change was modified by the neighborhood food environment among girls only. Future policy changes should incorporate consideration of ways to mitigate potentially inequitable geographic distribution of the health benefits of policy changes.

## Background

Childhood obesity prevalence is high in the United States, with even higher rates for children living in low-income households [1, 2]. Childhood obesity is associated with elevated risk of obesity in adulthood, and chronic health conditions to which obesity contributes [3–5]. Research suggests that environmental factors, such as the neighborhood built environment, may contribute to the establishment of healthy eating and physical activity behaviors, both of which influence obesity risk [6, 7].

The food environment, part of the built environment, is a complex set of environmental variables including factors like information about foods and their costs, proximity and access to food outlets, and the nutritional value of the foods available, which may all contribute to the foods purchased and consumed. The food environment is often quantified as the types and densities of food outlets available in neighborhoods, and associations have been identified between these variables and the types of food purchased [8–10] and children’s dietary behaviors [7, 11].

The Special Supplemental Nutrition Program for Women, Infants and Children (WIC) is a federal nutrition assistance program that provides a range of services, including nutrition education and supplemental foods, to pregnant and post-partum women, infants and children under the age of 5 living in households below 185% of the federal poverty level (FPL) [12]. In 2009, WIC supplemental food packages were revised to bring the contents in line with the Dietary Guidelines for Americans [13] and address the elevated prevalence of childhood obesity among WIC participants [2]. Improvements in the neighborhood availability of healthy foods have been reported following the food package change [14]. Because WIC participants live in low-income households, and low socioeconomic status limits geographic mobility [15], the diets of WIC participants may be especially impacted by the quality of foods (i.e. the availability of fresh fruits and vegetables) offered in the stores closest to their residence. Research has suggested that children living in adverse neighborhood environments may be less likely to benefit from behavioral interventions, which may lead to increases in health inequalities following interventions that do not take neighborhood factors into account [16].

The WIC food package change has been found to be associated with reduced obesity risk among WIC-participating children in Los Angeles County [17]. This reduced obesity risk could have a substantial population-level health impact given that nearly half of all children in the United States participate in WIC at some point during their first 5 years of life [12]. Moreover, previous research in the WIC population of Los Angeles County identified associations between neighborhood density of healthy food outlets and childhood adiposity [18], and between the 2009 WIC food package changes and improvements in diets [19]. The current study was conducted to evaluate whether the reduction in obesity risk associated with the WIC food package change in this population was modified by the food environment around the children’s residences. It was hypothesized that the reduction in obesity risk associated with the food package change would be A) stronger in neighborhoods with higher density of healthy food outlets, B) weaker in neighborhoods with higher density of unhealthy food outlets, and C) the modifying effect of healthy food outlet density would vary by neighborhood unhealthy food outlet density.

## Methods

### Subjects

Administrative data, collected during WIC participant eligibility certification and annual re-certification between 2003 and 2016, were used in this study. Data are compiled and managed as part of the Data Mining Project [20] by Public Health Foundation Enterprises (PHFE) WIC in Los Angeles County, California. WIC-participating children who received both the old and the new food package, who were not enrolled within 42 days of birth through age 4 (inclusive), who did not have a length (or height) and weight measurement each year (5 or more total measurements per child), and who did not have a census tract of residence recorded for each length (or height) and weight measurement were excluded from the sample. This was done to ensure that census tract of residence was known for every child each year during their WIC enrollment. To ensure confidentiality, only children living in census tracts with more than 5 WIC-participating children were included in the analysis (*N* = 148,634).

### Exposure

The exposure of interest was the food package received by children enrolled in WIC. Children who participated in WIC exclusively before October 1, 2009 received the *old food package*, and children who participated in WIC exclusively after that date received the *new food package*.

### Outcome

Child length (or height) and weight were measured at each eligibility certification and recertification by WIC staff. Obesity at age 4, defined as a body mass index (BMI)-for-age ≥ 95th percentile, was the outcome for this study. BMI-for-age was calculated based on height and weight using CDC growth curves [21]. Initial adiposity at enrollment was characterized with weight-for-length z-scores (WHZ), calculated based on the first length and weight measurements using CDC growth curves [21]. Length (or height) and weight measurements collected by WIC staff have high validity [22].

### Effect modifier

The food environment was identified as a potential effect modifier of the impact of the WIC package change on childhood obesity. The National Establishment Time-Series (NETS) was the source of food environment data [23]. The food environment was quantified as the density (count per square mile) of healthy (chain and independent grocery stores, fruit/vegetable vendors, and supermarkets) and unhealthy (chain and independent convenience stores, fast food, and liquor stores) food outlets in 2010 census tracts plus a 0.5-mile buffer around the border of the census tracts. This geographic unit has been previously identified as adequate to detect an association between the neighborhood food environment and obesity in the Los Angeles County WIC population [18]. In Los Angeles, liquor stores often sell unhealthy food items in addition to liquor, so the groupings for healthy and unhealthy food outlets were created to reflect the ratio of healthy to unhealthy foods available at each type of outlet [24]. Food environment data were available each year between 2003 and 2013. Each child’s food environment exposure was defined as the average density of healthy and unhealthy food outlets in the neighborhood of residence (census tract + 0.5-mile buffer) across the years of that child’s WIC enrollment.

### Covariates

Covariates available for inclusion in this analysis were child sex and race/ethnicity as reported by the child’s caregiver (Asian, African American, white, Hispanic, other), maternal education (<high school, high school degree, >high school) and language preference (English, Spanish, other), and categorical family income relative to the FPL (< 50% FPL, 50–100% FPL, > 100% FPL). Neighborhood contextual variables were obtained for the children’s census tract of residence in Los Angeles County from the American Community Survey (5-year estimates; old package years 2005–2009, new package years 2010–2014) and the 2000 and 2010 census [25, 26]. Contextual covariates included percent of residents in a neighborhood reporting having at least a high school education, percent of households with an income < 100% FPL, and percent of residents identifying as non-white. Population density in neighborhoods was calculated as the number of residents per square mile.

### Analysis

Characteristics of children receiving the new and old WIC packages and of their neighborhood of residence were summarized with frequencies, means and standard deviations. Associations between WIC package and obesity at age 4 were evaluated using Poisson regression models with robust standard errors for risk ratio estimation [27], accommodating clustering in census tracts [28]. Models were stratified by child sex and adjusted for child race/ethnicity and initial weight status (WHZ), maternal education and language preference, household income, and neighborhood characteristics including population density, the percent of residents with a high school education, the percent of residents with a household income < 100% FPL, and the percent of residents who were non-white. Polynomial terms (linear, quadratic and cubic) for food environment density variables (healthy and unhealthy) were included in preliminary models and interacted with each other. Models were reduced hierarchically by removing non-significant food environment interaction and main effects, with density of healthy food outlets (linear and quadratic), density of unhealthy food outlets (linear), and two-way interactions between healthy and unhealthy densities retained in the models. Hypotheses about the modification of the effect of the WIC food package change on obesity by the food environment were evaluated with interaction terms between the WIC package received (new vs. old) and food environment variables, specifically density (linear and quadratic) of healthy food outlets for Hypothesis A and density (linear) of unhealthy food outlets for Hypothesis B. Hypothesis C was evaluated with three-way interaction terms between WIC package and the two-way interactions between densities of healthy (linear and quadratic) and unhealthy (linear) food outlets. Risk ratios are presented at the 10th, 25th, 50th, 75th and 90th percentiles of healthy food outlet density (1.0, 1.5, 2.5, 4.0, and 6.5 healthy outlets per square mile) and unhealthy food outlet density (4.0, 6.0, 8.5, 12.0, and 16.5 unhealthy outlets per square mile). All analyses were conducted using SAS 9.4 software. *P*-values below 0.05 were considered statistically significant.

## Results

The final sample included 148,634 children, 57.3% of whom received the old WIC food package. Characteristics of WIC-participating children included in this study are presented in Table 1. Most children in both groups were Hispanic (89%) and lived in neighborhoods that were majority non-white (89%). More mothers and adult residents in neighborhoods of children receiving the new food package received at least a high school education (49 and 62% for mothers and adult neighborhood residents, respectively) than those of children receiving the old food package (37 and 59% for mothers and adult residents, respectively). Very low household income (< 50% FPL) was more prevalent among children receiving the new food package (28%) than among children receiving the old food package (19%). Children receiving the new food package lived in neighborhoods with a higher percent of residents living below the FPL (26%) than children receiving the old food package (22%). Maternal preference for speaking Spanish was lower among children enrolled in the new WIC package (49%) compared to children enrolled in the old package (65%). Initial WHZ and neighborhood population density were lower among boys and girls who received the new WIC food package than among those who received the old WIC food package.

**Table 1:** Characteristics of WIC participating children in Los Angeles County, California (*N* = 148,634)

Individual Characteristics	Boys		Girls	
	Old Package *N* = 43,546	New Package *N* = 32,195	Old Package *N* = 41,686	New Package *N* = 31,207
Initial WHZ^a^, mean ± SD	0.49 ± 1.51	0.37 ± 1.63	0.47 ± 1.86	0.37 ± 1.52
Age (y) at initial WHZ, mean ± SD	0.30 ± 0.35	0.27 ± 0.32	0.30 ± 0.35	0.28 ± 0.32
Obese at age 4 (y), n (%)	9098 (20.89)	5835 (18.12)	7250 (17.39)	5092 (16.32)
Race/ethnicity, n (%)				
Asian	1646 (3.79)	946 (2.94)	1538 (3.70)	903 (2.89)
African American	2003 (4.62)	1575 (4.89)	1857 (4.47)	1573 (5.04)
Hispanic	38,473 (88.67)	28,396 (88.21)	36,976 (89.03)	27,504 (88.15)
White	1218 (2.81)	738 (2.29)	1113 (2.68)	674 (2.16)
Other	51 (0.12)	535 (1.66)	46 (0.11)	547 (1.75)
Language Preference, n (%)				
English	13,887 (31.89)	15,703 (48.77)	13,431 (32.22)	15,084 (48.34)
Spanish	28,539 (65.54)	15,770 (48.98)	27,279 (65.44)	15,456 (49.53)
Other	1120 (2.57)	722 (2.24)	976 (2.34)	667 (2.14)
Maternal Education, n (%)				
< HS degree	27,250 (62.58)	16,300 (50.63)	26,161 (62.76)	15,885 (50.90)
HS degree	12,265 (28.17)	11,481 (35.66)	11,720 (28.11)	11,020 (35.31)
> HS degree	4031 (9.26)	4414 (13.71)	3805 (9.13)	4302 (13.79)
Household Income, n (%)				
< 50% FPL	8237 (18.92)	9121 (28.33)	8073 (19.37)	8730 (27.97)
50–100% FPL	21,544 (49.47)	15,471 (48.05)	20,558 (49.32)	15,164 (48.59)
> 100% FPL	13,765 (31.61)	7603 (23.62)	13,055 (31.32)	7313 (23.43)
**Neighborhood Characteristics**^b^				
Healthy outlets^c^ per sq. mi, mean ± SD	3.33 ± 2.54	2.99 ± 2.39	3.33 ± 2.54	2.97 ± 2.39
Unhealthy outlets^d^ per sq. mi, mean ± SD	9.97 ± 6.50	9.46 ± 5.73	9.97 ± 6.47	9.41 ± 5.69
Poverty percent, mean ± SD	22.60 ± 11.17	25.93 ± 11.55	22.65 ± 11.15	25.92 ± 11.63
Minority percent, mean ± SD	89.56 ± 14.02	89.66 ± 13.73	89.54 ± 14.01	89.63 ± 13.84
HS grad percent, mean ± SD	58.54 ± 15.74	62.04 ± 15.36	58.52 ± 15.70	62.07 ± 15.42
Residents per sq. mi, mean ± SD	18,297 ± 11,910	17,922 ± 11,894	18,295 ± 11,907	17,836 ± 11,790

*FPL* federal poverty level, *HS* high school, *MI* mile, *SD* standard deviation, *SQ* square, *WIC* Special Supplemental Nutrition Program for Women, Infants and Children, *WHZ* weight-for-height z-score, *y* years

^a^Initial WHZ corresponds to the first length and weight measurement for each participant

^b^Neighborhood was defined as the census tract of residence + a 0.5-mile buffer for food environment variables and as the census tract of residence for social environment variables

^c^Healthy outlets included supermarkets, chain and independent grocery stores and fruit/vegetable vendors

^d^Unhealthy outlets included fast food, liquor stores and chain and independent convenience stores

**Fig. 1:**
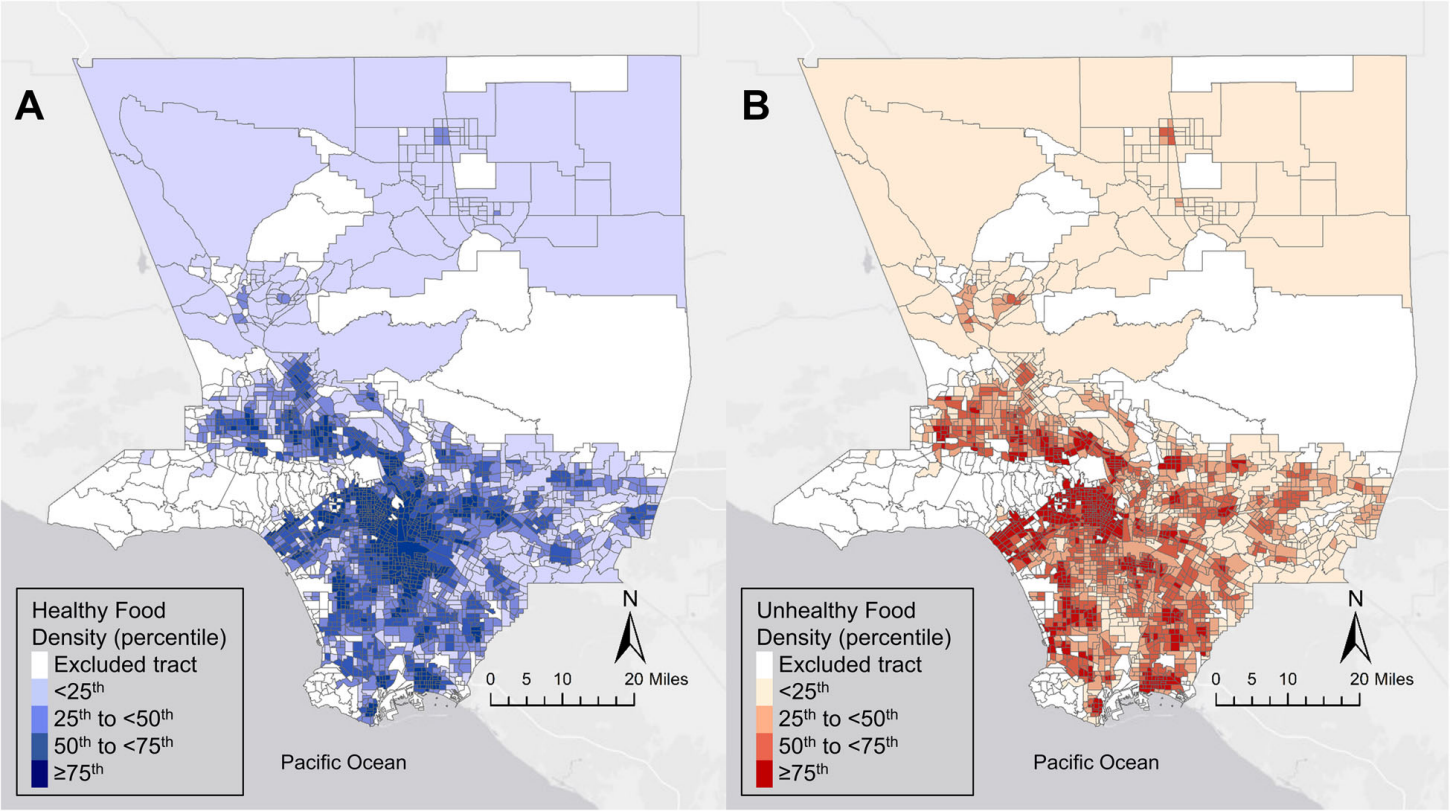
Quartiles for average density of healthy^1^ and unhealthy^2^ food outlets in 2010 census tracts with a 0.5-mile buffer of WIC-participants in Los Angeles County, California, 2002–2013.^3^. **a** Quartiles of healthy^1^ food outlet density per square mile. **b** Quartiles of unhealthy^2^ food outlet density per square mile.^1^ Healthy outlets included chain and independent grocers, fruit/vegetable vendors, and supermarkets. ^2^ Unhealthy outlets included chain and independent convenience stores, fast food, and liquor stores.^3^ The authors created the map for this publication using ArcGIS 10.3 (Environmental Systems Research Institute, Redlands, CA, USA)

The average density of unhealthy food outlets (9–10 per square mile) was much higher than the average density of healthy food outlets (3 per square mile), and children who received the new WIC food package lived in neighborhoods that had lower density of both healthy and unhealthy food outlets than children receiving the old WIC food package (Table 1). Healthy and unhealthy food outlet density categories were cross-tabulated for neighborhoods in Los Angeles County where study children lived (Table 2). Higher densities of healthy (Fig. 1a) and unhealthy (Fig. 1b) food outlets occurred in the same neighborhoods within the county (Pearson correlation coefficient 0.63, *p*-value < 0.0001). The combinations of healthy and unhealthy food outlet densities that were the least frequent were the highest healthy category and lowest unhealthy category (1.1% of included neighborhoods) and the lowest healthy category and highest unhealthy category (0.4% of included neighborhoods) (Table 2).

**Table 2:** WIC-participating study subjects and children under the age of 5 living in census tracts with different combinations of healthy and unhealthy food outlet densities in Los Angeles County, California ^a^

Food outlet density per sq. mile		Included Census Tracts *N* = 2080	Included WIC participants *N* = 148,634	Children living in Los Angeles County ^b^ *N* = 609,835
Healthy ^c^	Unhealthy ^d^	N (%)	N (%)	N (%)
0.0 to < 1.5	0.0 to < 6.0	254 (12.21)	10,209 (6.87)	73,781 (12.10)
1.5 to < 2.5	0.0 to < 6.0	104 (5.00)	6603 (4.44)	29,600 (4.85)
2.5 to < 4.0	0.0 to < 6.0	58 (2.79)	5194 (3.49)	17,866 (2.93)
4.0 to 21.1	0.0 to < 6.0	23 (1.11)	2586 (1.74)	7574 (1.24)
0.0 to < 1.5	6.0 to < 8.5	122 (5.87)	5047 (3.40)	33,623 (5.51)
1.5 to <2.5	6.0 to < 8.5	209 (10.05)	12,599 (8.48)	61,180 (10.03)
2.5 to < 4.0	6.0 to < 8.5	136 (6.54)	12,692 (8.54)	44,403 (7.28)
4.0 to 21.1	6.0 to < 8.5	78 (3.75)	10,177 (6.85)	27,786 (4.56)
0.0 to < 1.5	8.5 to < 12.0	45 (2.16)	1941 (1.31)	12,373 (2.03)
1.5 to <2.5	8.5 to < 12.0	157 (7.55)	7922 (5.33)	44,435 (7.29)
2.5 to < 4.0	8.5 to < 12.0	210 (10.10)	17,692 (11.90)	67,161 (11.01)
4.0 to 21.1	8.5 to < 12.0	142 (6.83)	19,051 (12.82)	51,991 (8.53)
0.0 to < 1.5	12.0 to 63.3	9 (0.43)	45 (0.03)	1677 (0.27)
1.5 to <2.5	12.0 to 63.3	56 (2.69)	1872 (1.26)	13,356 (2.19)
2.5 to < 4.0	12.0 to 63.3	145 (6.97)	6920 (4.66)	37,303 (6.12)
4.0 to 21.1	12.0 to 63.3	332 (15.96)	28,040 (18.87)	85,726 (14.06)

*SQ* square, *WIC* Special Supplemental Nutrition Program for Women, Infants and Children

^a^Categories of healthy and unhealthy food outlet density were determined by quartiles of the distribution for each variable

^b^Children under the age of 5 from the 2010 Census

^c^Healthy outlets included supermarkets, chain and independent grocery stores and fruit/vegetable vendors

^d^Unhealthy outlets included fast food, liquor stores and chain and independent convenience stores

**Fig. 2:**
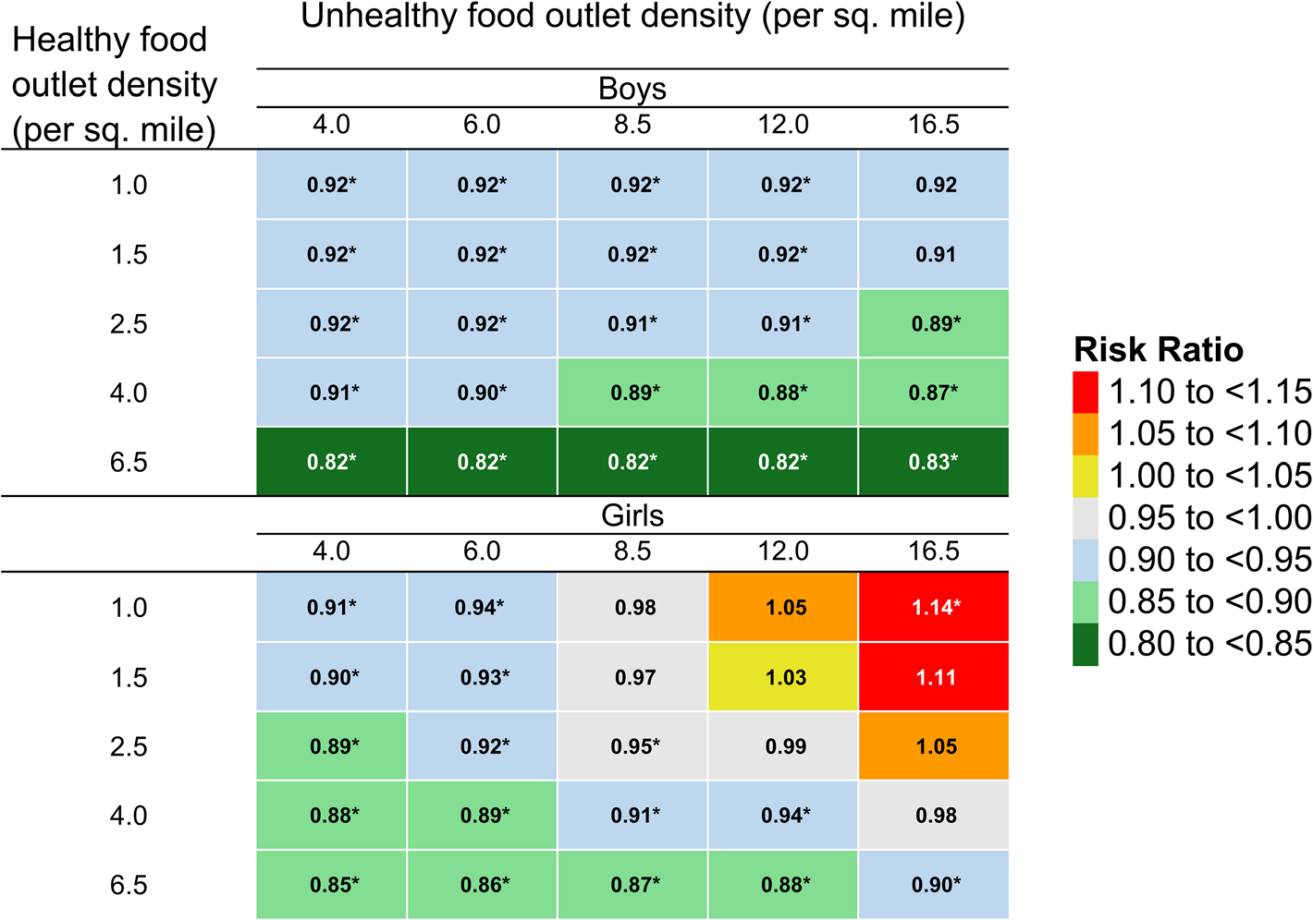
Heat map of the association between the new WIC food package and obesity at age 4 in boys and girls in Los Angeles County, California (*N* = 148,634) by density of healthy and unhealthy food outlets in neighborhood of residence.^1^. *Indicates statistically significant risk ratios. ^1^ Healthy outlets included chain and independent grocers, fruit/vegetable vendors, and supermarkets. Unhealthy outlets included chain and independent convenience stores, fast food, and liquor stores. Neighborhood was defined as the census tract of residence + a 0.5-mile buffer for food environment variables and as the census tract of residence for social environment variables. Risk ratios are from Poisson regression models adjusted for healthy food outlet density (linear and quadratic), unhealthy food outlet density (linear), interactions between healthy and unhealthy food outlet densities, child race, initial WHZ, age at last measurement, household income, maternal education and language preference, and neighborhood percent poverty, percent high school graduates, percent non-white and population density. The association between WIC package and obesity risk was assessed with 2-way interactions between WIC package and each food environment variable as well as 3-way interactions between WIC package and the 2-way interactions between healthy and unhealthy densities

In fully adjusted Poisson regression models – including food environment variables and interactions between the food package change and the food environment – significant associations were observed between the 2009 WIC food package changes and obesity risk at 4 years in nearly all neighborhoods, except those with the highest densities of unhealthy food outlets and lowest densities of healthy food outlets (Fig. 2, Supplemental Table 1). The food package change was associated with significant reductions in obesity risk at median healthy and unhealthy food environment densities (*RR* = 0.91, 95% CI = 0.88–0.95 for boys, and *RR* = 0.95, 95% CI = 0.91–0.98 for girls). The observed obesity risk reduction was more pronounced and more consistent across neighborhoods among boys than among girls. In neighborhoods with the healthiest food environments (highest density of healthy food outlets, lowest density of unhealthy food outlet density), the WIC food package change was associated with an 18% reduction in obesity risk (*RR* = 0.82, 95% CI = 0.76–0.90) among boys and a 15% reduction in obesity risk (*RR* = 0.85, 95% CI = 0.77–0.93) among girls.

For boys, risk ratios for the new WIC food package, compared to the old, indicated obesity risk reduction across the spectrum of healthy food outlet density in neighborhoods with ≤12 unhealthy outlets per square mile, with non-significant reductions observed in neighborhoods with higher unhealthy outlet densities (> 12 per square mile) and lower healthy outlet density (≤1.5 per square mile) (Fig. 2, Supplemental Table 1). Risk reductions associated with the WIC food package change for boys were larger at higher healthy food outlet densities, ranging from 0.92 (8% obesity risk reduction) in neighborhoods with 1.0 healthy food outlet per square mile to 0.82 (18% obesity risk reduction) in neighborhoods with 6.5 healthy food outlets per square mile; however, this effect-modification by the healthy food environment did not achieve statistical significance (hypothesis A: interaction *p*-value = 0.07). Risk reductions associated with the WIC food package change among boys were also not modified by the unhealthy food outlet density (hypothesis B: interaction *p*-value = 0.52), and were not jointly modified by healthy and unhealthy food outlet density among boys (hypothesis C: interaction *p*-value 0.16).

For girls, risk ratios for the new food package indicated reductions in obesity risk associated with the new food package, compared to the old, in neighborhoods with higher healthy food outlet density and lower unhealthy food outlet density (Fig. 2, Supplemental Table 1). Among girls living in neighborhoods above the median unhealthy outlet density (> 8.5 per square mile) and below the median healthy outlet density (< 2.5 per square mile), the WIC food package change was not associated with reduced obesity risk. The association between the WIC food package change and obesity among girls was not significantly modified by healthy food outlet density, with similar reductions in obesity risk observed across the range of healthy food outlet density (hypothesis A: interaction *p*-value = 0.72). Among girls, larger risk reductions were observed at lower densities of unhealthy food outlets: for girls in neighborhoods with 2.5 healthy outlets per square mile, the risk ratio was 0.89 at 4.0 unhealthy outlets per square mile but 1.05 at 16.5 unhealthy outlets per square mile. This modification of the effect of the WIC food package change by unhealthy food outlet density for girls was statistically significant (hypothesis B: interaction *p*-value = 0.004). Additionally, there was a suggestion of joint modification of WIC package effect by healthy and unhealthy food outlet densities. The modifying effect of healthy food outlet density on the association between WIC package and obesity risk at age 4 was stronger at higher unhealthy food outlet densities, though this did not achieve statistical significance (hypothesis C: interaction *p*-value = 0.09). For example, for girls living in neighborhoods with a low unhealthy outlet density (4.0 per square mile), the WIC package change was associated with a 9% obesity risk reduction at low healthy outlet density (1.0 per square mile), whereas at high healthy outlet density (6.5 per square mile), the WIC package change was associated with an obesity risk reduction of 15% (Fig. 2, Supplemental Table 1). In turn, for high unhealthy outlet density neighborhoods (16.5 per square mile), the WIC package change was associated with a 14% obesity risk *increase* at the low healthy outlet density (1.0 per square mile) and a 10% obesity risk reduction at high healthy outlet density (6.5 per square mile).

## Discussion

Among a sample of WIC-participating children in Los Angeles County (2003–2016), the 2009 WIC food package change was associated with a significant 9 and 5% reduction in the risk of obesity at age 4 among boys and girls, respectively, at the median healthy and unhealthy food environment densities after accounting for other contextual factors in the neighborhood of residence. In neighborhoods with the healthiest food environments (highest healthy food outlet density and lowest unhealthy food outlet density) the WIC food package change was associated with a significant 18 and 15% reduction in obesity risk among boys and girls, respectively. Associations between the new WIC food package (after 2009) and reductions in obesity risk were stronger for boys and girls living in neighborhoods with higher densities of healthy food outlets; however, healthy food outlet density did not significantly modify the association between the WIC food package change and childhood obesity. Density of unhealthy food outlets significantly modified the association between the WIC food package change and obesity among girls, with higher unhealthy food density associated with a weaker association between the food package change and reduction in obesity. The lack of a modifying effect by healthy food density was unexpected, but the modifying effect of unhealthy density conformed to our hypothesis that a weaker effect of the WIC food package change would be observed in neighborhoods with higher unhealthy food density.

Previous research has identified neighborhood contextual factors that modify the effects of interventions for obesity and related behaviors, though previous studies have focused on contextual modifiers in populations of children older than the WIC participants in this study. The built environment, including variables for food environment exposures, was found to modify the effect of family-based treatment for childhood obesity in four small randomized controlled trials involving children aged 8 to 12 years, with greater BMI z-score reductions observed for children living near more parkland, fewer convenience stores, and fewer supermarkets [29]. Fewer convenience stores being associated with greater BMI z-score reduction is comparable to the present study’s finding that the WIC food package association with reduced obesity risk was significantly stronger among girls living in neighborhoods with lower density of unhealthy food outlets; however, the relationship between fewer supermarkets and greater BMI z-score reduction is not consistent with the lack of effect modification by healthy food outlet density in the present study. In a randomized trial of practice-based interventions for childhood obesity in children from 6 to 12 years of age, distance to the nearest supermarket modified the association between the intervention and increased fruit and vegetable intake and reduced BMI z-score, with shorter distance to the supermarket being associated with stronger effects for both outcomes [30]. Similarly, in the present study, the effect of the WIC food package changes was stronger among children living in neighborhoods with higher density of healthy food outlets, though the interaction term measuring effect modification did not achieve statistical significance. Neighborhood crime moderated the increase in physical activity observed in a randomized study of an intensive physical activity intervention among 6 to 10 year old children, with higher crime associated with significantly smaller physical activity increases [16], which while not directly comparable to the present study reflects a similar framework in which a neighborhood contextual variable (i.e. crime or unhealthy food density) leads to reduced benefits from an intervention (i.e. a smaller physical activity increase or a smaller obesity reduction).

The absence of significant effect modification by the density of healthy food outlets was unexpected. Prior research identified a significant association between the density of healthy food outlets in neighborhoods and childhood adiposity [18]; however, while the effect of the WIC food package change was observed to be stronger in neighborhoods with higher densities of healthy food outlets, this did not achieve statistical significance. A study of neighborhood and parental influences on diet behavior conducted among African American and Hispanic caregivers of children between 2 and 5 years of age found that while neighborhood contextual factors (i.e. lack of healthy food outlets) may present barriers to preferred child diets, parents are able to exercise tremendous agency in implementing strategies to mitigate the health limiting influences of neighborhoods [31]. The nutrition education efforts by WIC may be integral to parents’ efforts to establish healthy dietary practices in their children [32]. This education provided to all WIC participating caregivers may reduce the moderating influence of the density of healthy foods outlets in neighborhood of residence.

Our study found that the density of unhealthy food outlets modified the relationship between the WIC food package change and obesity among girls, but not among boys, with a smaller obesity reduction observed at higher densities of unhealthy food outlets. This is in agreement with prior research on food environments around schools in California which found stronger associations between fast food density and overweight among girls than among boys [6]. The reason the effect of the WIC package change on obesity was modified by unhealthy food environment density only among girls is unclear; further research is merited as a number of studies have found stronger neighborhood effects on adiposity among girls [6, 33].

The density of healthy and unhealthy food outlets around the residence may modify the relationship between the WIC food package change and childhood obesity through its influence on diet quality. There is mixed evidence on the relationship between the food environment, including prices of and access to foods, and food purchasing behaviors [34, 35]. In a recent study of WIC-participating women, there was no association between distance to the nearest grocery store or supermarket and redemption of the cash value vouchers for fruits and vegetables [36]. However, previous research has found that individuals who live closer to outlets that sell fresh produce consume more fruits and vegetables [37] and individuals who live in proximity to unhealthy food outlets consume fewer fruits and vegetables [38]. Living in a neighborhood with an abundance of food outlets providing unhealthy items may encourage the consumption of high calorie and low nutritional value items, like sugar-sweetened beverages, among nutrition assistance program participants [39]. Similarly, living further from supermarkets or other food outlets that sell fresh fruits and vegetables may contribute to lower consumption of produce among food assistance program participants compared to non-participating individuals [39].

Better diet quality has been reported among children enrolled in WIC after, compared to before, the 2009 food package change [40]. A study conducted in Wisconsin following the implementation of vouchers for fresh fruits and vegetables found that only 45% of WIC participants were redeeming the full dollar amount and another 32% part of the dollar amount, implying that 23% of participants were not redeeming any of the voucher value [41]. A more recent study found that only 63% of WIC participating women in Jefferson County, Alabama, were regular redeemers of the vouchers, and that regular redeemers were more likely to purchase fruits and vegetables at grocery stores and consumed more fruits and vegetables than participants who irregularly or never redeemed the vouchers [36]. The provision of vouchers alone will not increase the consumption of fresh fruits and vegetables if individuals are unable to find a store in which to purchase fruits and vegetables.

The lack of modification of the association between the WIC food package change and obesity by healthy food density is consistent with multiple explanations: either the amount of healthier foods consumed by WIC participants in Los Angeles County is not constrained by the density of stores selling them, or alternatively WIC supplemental food packages and nutrition education successfully mitigates the impact of limitations in the availability of stores that sell healthy foods. The association between the 2009 WIC food package change and obesity reduction was significantly weaker for girls who lived in neighborhoods with higher densities of unhealthy food outlets. Therefore, the change of the WIC food package, while associated with improved diets of WIC-participating children generally [19], did not benefit all children equally. The abundance of unhealthy food options in some neighborhoods was sufficient to obscure the benefits of the new food package, and additional nutrition educational efforts for children who live in food swamps (i.e. neighborhoods with high density of unhealthy food outlets) may be needed. Gender differences in the associations between the 2009 WIC food package change and childhood obesity have been reported previously [17, 42], and more detailed research may be needed to evaluate why benefits of the food package changes seem to have been more consistent for boys than for girls.

This study has notable strengths. The Los Angeles County WIC-population is a large and well-characterized sample. Longitudinal data were available for individuals and neighborhoods, which allowed us to quantify the food environment exposure over the 5 years of each child’s enrollment in WIC. All reported associations were adjusted for healthy and unhealthy food environment densities; neighborhood characteristics including population density and socioeconomic status indicators; and family/individual characteristics that may contribute to obesity risk. Food environment data was only available through 2013, and misclassification of food environment exposure may have occurred due to changes neighborhood food outlets for children participating between 2014 and 2016. The uncertain geographic context problem, namely the use of a geographic context (i.e. census tract plus buffers for residential neighborhood) which may not reflect the true geographic context that influences the health behavior or outcome being studied [43], is also a limitation. However, this uncertainty and the misclassification of food environment exposure that may result from it should be independent of, and non-differential, with respect to outcome (obesity) and exposure (WIC food package), although this does not guarantee any bias introduced to measures of association will be towards null values [44]. The administrative data used in this study contain no information on diet, which disallowed the assessment of the relationship between the food environment and diet in these children. The sample in this study was predominantly Hispanic and live in one large county in Southern California, so the generalizability of the results may be limited.

## Conclusions

The 2009 WIC food package change was associated with significant reductions in obesity in neighborhoods across the distribution of healthy and unhealthy food outlet densities. However, the obesity reduction was significantly weaker for girls who lived in neighborhoods with higher densities of unhealthy food outlets. Mechanisms for gender differences in effect modification by the food environment are unknown, but these merit further study. Nutrition assistance programs like WIC may need to consider tailoring educational efforts to provide all participants with information about how to procure a healthy diet regardless of adverse neighborhood environments to ensure more equitable returns on future changes in the programs. WIC Authorized Vendors are a key partner of WIC and a key component of the healthy food environment, and it is important for WIC to continue to work closely with these vendors to create opportunities for participants to maximize their WIC food purchases.

## Supplementary information

**Additional file 1: Supplemental Table 1.** Risk ratio for obesity at age 4 in boys and girls in Los Angeles County, California (*N*=148,634) by density of healthy and unhealthy food outlets in neighborhood of residence^1^.

## Data Availability

Data used in this study are confidential and therefore not publicly available. Data cannot be shared by the authors according to an agreement between PHFE WIC and the California Department of Public Health WIC Division.

## Abbreviations

BMI: Body mass index
CI: Confidence interval
FPL: Federal poverty level
HS: High school
MI: Mile
NETS: National Establishment Time-Series
PHFE: Public Health Foundation Enterprises
RR: Risk ratio
SD: Standard deviation
SQ: Square
WHZ: Weight-for-height z-score
WIC: The Special Supplemental Nutrition Program for Women, Infants and Children
Y: Years
