# Supplementary material for: The neighborhood food environment modifies the effect of the 2009 WIC food package change on childhood obesity in Los Angeles County, California
Source: BMC Public Health. 2020 May 13;20:678. doi: 10.1186/s12889-020-08779-2 (PMC7222567; doi:10.1186/s12889-020-08779-2)
Supplement: Supplementary file 1 — Additional file 1: Supplemental Table 1. Risk ratio for obesity at age 4 in boys and girls in Los Angeles County, California (N=148,634) by density of healthy and unhealthy food outlets in neighborhood of residence1. [file 12889_2020_8779_MOESM1_ESM.docx]

**Supplemental Table 1** Risk ratio for obesity at age 4 in boys and girls in Los Angeles County, California (N=148,634) by density of healthy and unhealthy food outlets in neighborhood of residence.^1^

| Healthy food outlet density |  | Unhealthy food outlet density | | | | | | | | | | | | | |
| --- | --- | --- | --- | --- | --- | --- | --- | --- | --- | --- | --- | --- | --- | --- | --- |
|  |  | Boys | | | | | | | | | | | | | |
|  | Food package | 4.0 per sq. mile | |  | 6.0 per sq. mile | |  | 8.5 per sq. mile | |  | 12.0 per sq. mile | |  | 16.5 per sq. mile | |
|  |  | RR | 95% CI |  | RR | 95% CI |  | RR | 95% CI |  | RR | 95% CI |  | RR | 95% CI |
| 1.0 per sq. mile | New | **0.92** | **0.87, 0.97** |  | **0.92** | **0.88, 0.96** |  | **0.92** | **0.88, 0.96** |  | **0.92** | **0.85, 0.99** |  | 0.92 | 0.82, 1.03 |
|  | Old | 1.00 | Ref. |  | 1.00 | Ref. |  | 1.00 | Ref. |  | 1.00 | Ref. |  | 1.00 | Ref. |
| 1.5 per sq. mile | New | **0.92** | **0.88, 0.97** |  | **0.92** | **0.89, 0.96** |  | **0.92** | **0.88, 0.96** |  | **0.92** | **0.86, 0.97** |  | 0.91 | 0.82, 1.01 |
|  | Old | 1.00 | Ref. |  | 1.00 | Ref. |  | 1.00 | Ref. |  | 1.00 | Ref. |  | 1.00 | Ref. |
| 2.5 per sq. mile | New | **0.92** | **0.88, 0.97** |  | **0.92** | **0.88, 0.96** |  | **0.91** | **0.88, 0.95** |  | **0.91** | **0.87, 0.95** |  | **0.89** | **0.83, 0.96** |
|  | Old | 1.00 | Ref. |  | 1.00 | Ref. |  | 1.00 | Ref. |  | 1.00 | Ref. |  | 1.00 | Ref. |
| 4.0 per sq. mile | New | **0.91** | **0.85, 0.97** |  | **0.90** | **0.85, 0.95** |  | **0.89** | **0.85, 0.93** |  | **0.88** | **0.85, 0.92** |  | **0.87** | **0.83, 0.92** |
|  | Old | 1.00 | Ref. |  | 1.00 | Ref. |  | 1.00 | Ref. |  | 1.00 | Ref. |  | 1.00 | Ref. |
| 6.5 per sq. mile | New | **0.82** | **0.76, 0.90** |  | **0.82** | **0.76, 0.89** |  | **0.82** | **0.77, 0.88** |  | **0.82** | **0.78, 0.87** |  | **0.83** | **0.78, 0.87** |
|  | Old | 1.00 | Ref. |  | 1.00 | Ref. |  | 1.00 | Ref. |  | 1.00 | Ref. |  | 1.00 | Ref. |
|  |  | Girls | | | | | | | | | | | | | |
|  |  | 4.0 per sq. mile | |  | 6.0 per sq. mile | |  | 8.5 per sq. mile | |  | 12.0 per sq. mile | |  | 16.5 per sq. mile | |
|  |  | RR | 95% CI |  | RR | 95% CI |  | RR | 95% CI |  | RR | 95% CI |  | RR | 95% CI |
| 1.0 per sq. mile | New | **0.91** | **0.86, 0.96** |  | **0.94** | **0.90, 0.99** |  | 0.98 | 0.93, 1.04 |  | 1.05 | 0.97, 1.14 |  | **1.14** | **1.00, 1.29** |
|  | Old | 1.00 | Ref. |  | 1.00 | Ref. |  | 1.00 | Ref. |  | 1.00 | Ref. |  | 1.00 | Ref. |
| 1.5 per sq. mile | New | **0.90** | **0.86, 0.95** |  | **0.93** | **0.89, 0.97** |  | 0.97 | 0.93, 1.01 |  | 1.03 | 0.96, 1.10 |  | 1.11 | 0.99, 1.23 |
|  | Old | 1.00 | Ref. |  | 1.00 | Ref. |  | 1.00 | Ref. |  | 1.00 | Ref. |  | 1.00 | Ref. |
| 2.5 per sq. mile | New | **0.89** | **0.84, 0.94** |  | **0.92** | **0.87, 0.96** |  | **0.95** | **0.91, 0.98** |  | 0.99 | 0.94, 1.04 |  | 1.05 | 0.97, 1.13 |
|  | Old | 1.00 | Ref. |  | 1.00 | Ref. |  | 1.00 | Ref. |  | 1.00 | Ref. |  | 1.00 | Ref. |
| 4.0 per sq. mile | New | **0.88** | **0.81, 0.94** |  | **0.89** | **0.84, 0.95** |  | **0.91** | **0.87, 0.96** |  | **0.94** | **0.90, 0.98** |  | 0.98 | 0.92, 1.04 |
|  | Old | 1.00 | Ref. |  | 1.00 | Ref. |  | 1.00 | Ref. |  | 1.00 | Ref. |  | 1.00 | Ref. |
| 6.5 per sq. mile | New | **0.85** | **0.77, 0.94** |  | **0.86** | **0.79, 0.93** |  | **0.87** | **0.81, 0.93** |  | **0.88** | **0.83, 0.94** |  | **0.90** | **0.84, 0.95** |
|  | Old | 1.00 | Ref. |  | 1.00 | Ref. |  | 1.00 | Ref. |  | 1.00 | Ref. |  | 1.00 | Ref. |

CI=confidence interval; Ref=reference; RR=risk ratio; SQ=square; WIC=Special Supplemental Nutrition Program for Women, Infants and Children

^1^ Neighborhood was defined as the census tract of residence + a 0.5-mile buffer for food environment variables and as the census tract of residence for social environment variables. Risk ratios and confidence intervals are from Poisson regression models adjusted for healthy food outlet density (linear and quadratic), unhealthy food outlet density (linear), interactions between healthy and unhealthy food outlet densities, child race, initial WHZ, age at last measurement, household income, maternal education and language preference, and neighborhood percent poverty, percent high school graduates, percent non-white and population density. The association between WIC package and obesity risk was assessed with 2-way interactions between WIC package and each food environment variable as well as 3-way interactions between WIC package and the 2-way interactions between healthy and unhealthy densities.
